# Oscillations in U.S. COVID-19 Incidence and Mortality Data Reflect Diagnostic and Reporting Factors

**DOI:** 10.1128/mSystems.00544-20

**Published:** 2020-07-14

**Authors:** Aviv Bergman, Yehonatan Sella, Peter Agre, Arturo Casadevall

**Affiliations:** a Department of Systems and Computational Biology, Albert Einstein College of Medicine, Bronx, New York, USA; b Department of Molecular Microbiology and Immunology, Johns Hopkins School of Public Health, Baltimore, Maryland, USA; Duke University School of Medicine

**Keywords:** COVID-19, coronavirus, epidemiology

## Abstract

The coronavirus disease 2019 (COVID-19) pandemic currently in process differs from other infectious disease calamities that have previously plagued humanity in the vast amount of information that is produced each day, which includes daily estimates of the disease incidence and mortality data. Apart from providing actionable information to public health authorities on the trend of the pandemic, the daily incidence reflects the process of disease in a susceptible population and thus reflects the pathogenesis of COVID-19, the public health response, and diagnosis and reporting. Both new daily cases and daily mortality data in the United States exhibit periodic oscillatory patterns. By analyzing New York City (NYC) and Los Angeles (LA) testing data, we demonstrate that this oscillation in the number of cases can be strongly explained by the daily variation in testing. This seems to rule out alternative hypotheses, such as increased infections on certain days of the week, as driving this oscillation. Similarly, we show that the apparent oscillation in mortality in the U.S. data are mostly an artifact of reporting, which disappears in data sets that record death by episode date, such as the NYC and LA data sets. Periodic oscillations in COVID-19 incidence and mortality data reflect testing and reporting practices and contingencies. Thus, these contingencies should be considered first prior to suggesting biological mechanisms.

**IMPORTANCE** The incidence and mortality data for the COVID-19 data in the United States show periodic oscillations, giving the curve a distinctive serrated pattern. In this study, we show that these periodic highs and lows in incidence and mortality data are due to daily differences in testing for the virus and death reporting, respectively. These findings are important because they provide an explanation based on public health practices and shortcomings rather than biological explanations, such as infection dynamics. In other words, when oscillations occur in epidemiological data, a search for causes should begin with how the public health system produces and reports the information before considering other causes, such as infection cycles and higher incidences of events on certain days. Our results suggest that when oscillations occur in epidemiological data, this may be a signal that there are shortcomings in the public health system generating that information.

## Abstract (precis)

The incidence and mortality data for the COVID-19 data in the United States show periodic oscillations, giving the curve a distinctive serrated pattern. In this study, we show that these periodic highs and lows in incidence and mortality data are due to daily differences in testing for the virus and death reporting, respectively. These findings are important because they provide an explanation based on public health practices and shortcomings rather than biological explanations, such as infection dynamics. In other words, when oscillations occur in epidemiological data, a search for causes should begin with how the public health system produces and reports the information before considering other causes, such as infection cycles and higher incidences of events on certain days. Our results suggest that when oscillations occur in epidemiological data, this may be a signal that there are shortcomings in the public health system generating that information.

## INTRODUCTION

Epidemic case incidence data provide raw information on the course and outcome of infectious disease outbreaks. Such data inform on whether the epidemic is surging, ebbing, or evolving into an endemic. Trends in the case incidence data provide insight into the efficacy of medical and behavioral interventions, such as vaccines and social distancing measures, respectively. In addition, trends in case incidence data as a function of time reflect biological parameters of the host-microbe interaction, including prevalence of disease among infected individuals, contagiousness of infection, diagnostic capacity, and the size of the susceptible population. Since the case incidence data must ultimately be gathered, compiled, and analyzed, the numbers also reflect the public health capacity and infrastructure available to track the course of the epidemic. Consequently, the case incidence data are numbers that reflect many variables, ranging from biological constraints of the host and pathogen entities to medical, diagnostic, public health, and social structures on the affected population.

Because case incidence data reflect the conglomerations of many different informational components with variable contributions, it is difficult to tease out their individual contributions from trend curves. Given the complexity of how epidemic case incidence data emerge, epidemiological modeling is a necessary and important tool for understanding how individual variables contribute to this number. However, a higher-order analysis of case incidence data can capture patterns that reflect the contribution of the individual contributory components.

For example, a prevalent feature of many COVID-19 case incidence and mortality data sets is the presence of weekly periodic oscillations, as shown for example by Ricon-Becker et al. (1), who used an auto-correlation analysis to establish this weekly periodicity in multiple data sets. This prompts the question of whether these oscillations are an effect of societal factors that impact the course of the epidemic, such as increased activity on certain days of the week, leading to spikes in infection, or whether it is a function of the tempo of diagnostic and reporting activity that is independent of the course of the epidemic. Similarly, with respect to oscillations in mortality data, one can ask whether these reflect true variations in quality of care by day of the week or mere artifacts of reporting. Finally, one can ask if the observed oscillations in case incidence data reflect any biological parameters, such as time to infectivity or time to symptom onset. Ricon-Becker et al. (1) hypothesized that the weekly periodicity reflects societal factors, such as increased intergenerational infections on weekends as well as variations in quality of care. On the other hand, an analysis of case incidence data in Germany revealed an oscillatory pattern that was attributed to weekly fluctuations in case reporting (2).

In this paper, we analyze the trends and periodicity in COVID-19 case incidence and mortality data in the United States, both in the country at large and specifically in New York City (NYC) and Los Angeles, CA (LA). We use a power spectrum analysis on the detrended data as opposed to an auto-correlation analysis. Power spectrum analysis using the Fourier transform is a standard method for analyzing periodicity of a time series by converting it to the frequency domain, although the application of this method to infectious diseases appears to be novel. We are also careful to identify data sets in which positive tests and deaths are back-dated to the episode date, rather than being dated to the reporting date, in order to control for the effect of reporting. We conclude from our analysis that the observed periodicity can be very well explained by the weekly tempo of testing and reporting and does not appear to significantly reflect other societal or biological factors.

## RESULTS AND DISCUSSION

**FIG 1:**
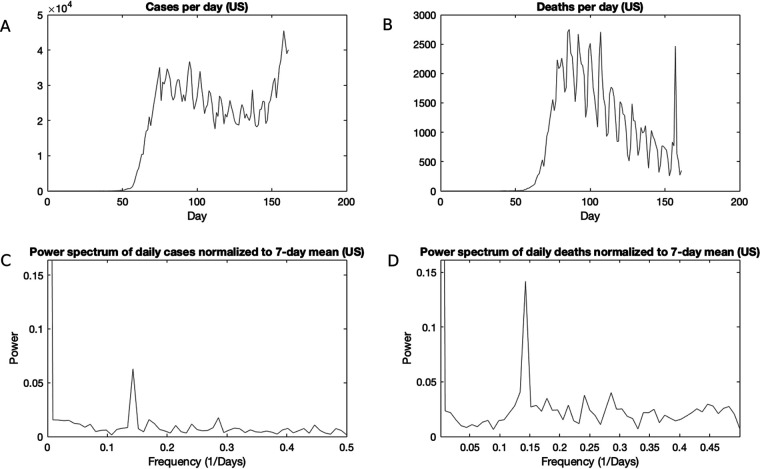
Periodic behavior in COVID-19 cases and deaths in the United States. (A) Daily cases in the United States over time; (B) daily deaths in the United States over time; (C) power spectrum of daily cases in the United States, normalized to a 7-day moving average; (D) power spectrum of daily deaths in the United States, normalize to a 7-day moving average.

To quantify the observed oscillation in incidence and mortality, we performed a power spectrum analysis of U.S. cases, which revealed a clear peak at the frequency of 0.143, corresponding to a period of 7.0 days (Fig. 1C). A similar analysis of NYC and LA cases revealed major periods of every 6.8 days and 6.9 days, respectively, and minor periods of every 3.5 days and 3.3 days, respectively (see Fig. S2A and C in the supplemental material). The presence of clear gaps between peaks in the power spectrum signifies periodic patterns as opposed to chaotic or stochastic signatures (3, 4). Mortality data in the United States also revealed a periodicity of 7.0 days (Fig. 1D).

The periodicities of 6.8, 6.9, and 7.0 days are very close to the length of a week, tracking with the results of Ricon-Becker et al. (1), who used an auto-correlation analysis. The weekly periodicity suggests that societal rather than purely biological factors are at play. The more minor periodicities of 3.5 and 3.3 may potentially arise artificially due to their nature as approximately 1/2 of a week. Ricon-Becker et al. (1) conjectured that this weekly periodicity is due to societal factors, such as increased weekend activity or variation in quality of care by day of the week. However, we investigated the more trivial potential explanation that the weekly periodicity reflects variation in diagnostic and reporting activity related to the tempo of the week, rather than true variation in incidence and mortality. For example, increased testing or greater reporting of cases and deaths on some days of the week as backlogs are cleared from the weekend may produce increases in reported cases and deaths that, repeated each week, will create a recurring periodicity.

**FIG 2:**
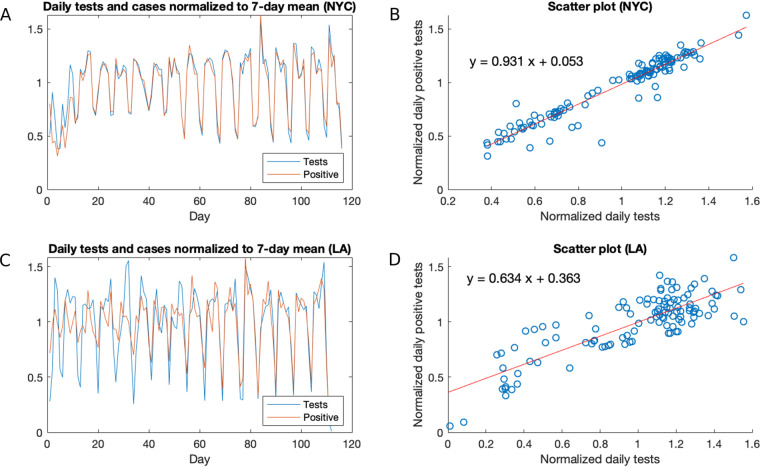
Analysis of daily cases and deaths in NYC and LA. (A) Daily tests and positive tests in NYC over time, normalized to a 7-day moving average; (B) scatterplot and regression line for the normalized tests and positive tests data in NYC; (C) daily tests and positive tests in LA over time, normalized to a 7-day moving average; (D) scatterplot and regression line for the normalized tests and positive tests data in LA.

To investigate the hypothesis that the oscillations in incidence are simply a reflection of the testing done, we focused on the data sets for NYC and LA, from which testing and incidence data were available and which also have the advantage of being localized regions with high incidences. The number of tests in NYC has a clear valley on Sundays and peaks on Wednesdays and Thursdays, and the number of positive tests exhibits the same pattern (Fig. S1A and B). Indeed, when numbers of both total tests and positive tests are normalized to a 7-day average, we observe a near perfect correlation between the two (*r* = 0.96) (Fig. 2A). Moreover, the two normalized time series align almost perfectly, as seen visually (Fig. 2B) or by the fact that the line *y* = *x* is close to the line of best fit (Fig. 1D). Data in LA yields similar, though less strong, results (*r* = 0.83) (Fig. 2C and D), probably due to lower numbers of cases initially giving rise to more noise, though the alignment between these normalized time series strengthens with time, as the volume of cases and tests increase. The strong correlation between cases and tests persists throughout the course of the epidemic, even as the testing capacity in New York and California has greatly increased over time. In New York, the general trends of testing and case incidence have diverged, as the testing consistently has increased, while the number of cases has fallen and flattened. Regardless, when controlling for these overall trends, the day-to-day variation in testing correlates strongly with the corresponding variation in numbers of positive tests, strongly suggesting that these day-to-day variations in cases are an artifact of testing. This suggests that alternative potential explanations, such as increased social activity on certain days of the week, leading to a greater infection rate, are not significant drivers of the observed oscillations.

10.1128/mSystems.00544-20.1 FIG S1 Analysis of reported tests and positive tests in NYC and LA. (A) Daily tests in NYC; (B) daily positive tests in NYC; (C) daily tests in LA; (D) daily positive tests in LA. Download FIG S1, TIF file, 0.3 MB. Copyright © 2020 Bergman et al. 2020 Bergman et al. This content is distributed under the terms of the Creative Commons Attribution 4.0 International license.

10.1128/mSystems.00544-20.2 FIG S2 Power spectra of the normalized time series of reported cases and the ratio of positive cases in NYC and LA. The first month of LA data was omitted due to low testing. (A) Power spectrum of the normalized reported cases in NYC; (B) power spectrum of the normalized ratio of positive tests in NYC, revealing no clear period; (C) power spectrum of the normalized reported cases in LA; (D) power spectrum of the normalized ratio of positive tests in LA, revealing no clear period. Download FIG S2, TIF file, 0.2 MB. Copyright © 2020 Bergman et al. 2020 Bergman et al. This content is distributed under the terms of the Creative Commons Attribution 4.0 International license.

To control for the effect of testing and investigate whether any residual periodicity remains, we analyzed the ratio of positive tests as a function of time. A power spectrum analysis of the resulting normalized time series in NYC and LA revealed no clear periodicity (Fig. S2B). While there is some periodicity at around 7 days, its power is comparable to that of a large range of frequencies, suggesting that weekly periodicity, if it exists, is drowned out by noise.

**FIG 3:**
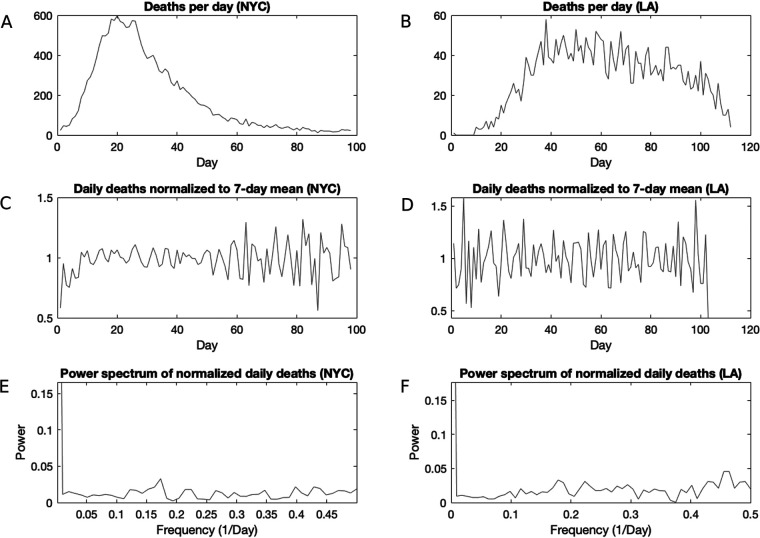
Analysis of mortality data in NYC and LA. (A) Daily deaths in NYC; (B) daily deaths in LA; (C) daily deaths in NYC, normalized to a 7-day moving average; (D) daily deaths in LA, normalized to a 7-day moving average; E) power spectrum of the normalized mortality data in NYC, revealing no clear period; (F) power spectrum of daily deaths in LA, revealing no clear period.

Similarly, the oscillations in mortality data appear to be artifacts of reporting. We note an important difference between the U.S. data and the data sets that we have obtained from NYC and LA: although in the U.S. data set, case and death dates reflect the reporting date, the NYC and LA data are back-dated to the episode date and are therefore more reliable indicators of the mortality and incidence time series. Having corrected for the episode date, we do not observe a weekly periodicity in NYC or LA deaths. When normalized to a 7-day moving average, the NYC and LA mortality data show some oscillation but no clear periodicity (Fig. 3C and D). Indeed, the power spectra of the normalized mortality data do not yield clear peaks (Fig. 3E and F), indicating a stochastic rather than a periodic signal.

Several studies have shown variations in medical care between weekdays and weekends. In this regard, patients having procedures later in the week and on weekends had higher mortality than those treated earlier in the week (5). Similarly, individuals admitted to hospitals during weekends have higher mortality than those admitted during the week (6). However, such variations in medical care do not appear to drive clear periodicity in NYC and LA mortality data.

Our analysis does not rule out the existence of societal factors, such as increased infections on weekends or variations in quality of care, it only demonstrates that such factors, to the extent that they exist, are sufficiently minor that they do not translate to clear signals. Increased infections on weekends, for example, may be smoothed out by the continuous infection dynamics, given the many stochastic components involved in these dynamics.

Regardless of the above caveats, we considered the possibility of biological causes for oscillation but were able to rule these out. The time required between infection and capacity to spread the virus was estimated to be 2.3 days (7). One might conjecture that this time delay will manifest in a minor periodicity of around 2.3 days. However, such a time delay will not lead to periodicity in incidence unless it is coupled with periodic bursts of increased interaction and infection, for example, as may be caused by increased activity on the weekend. However, our above analysis of NYC data, demonstrating a strong correlation with testing, ruled out increased infection rate as a significant driver of oscillation, making it unlikely that the time delay of 2.3 days manifests in periodicity in the data. Indeed, we do not identify a strong frequency of 2.3 in any of the power spectrum analyses. This reasoning led us away from any biological explanation of the apparent periodicities.

In summary, we observed periodic weekly oscillations in the incidence and mortality data for COVID-19. The oscillations in incidence data showed a very strong correlation with the day-to-day testing numbers, implying that these were directly caused by variation in testing. Mortality oscillations appear to reflect the weekly tempo of reporting, rather than true oscillations in deaths. Hence, oscillations of incidence and mortality data may point to biasing practices in case reporting, which should be considered first prior to suggesting social or biological mechanisms.

## MATERIALS AND METHODS

U.S. daily new case and death data were obtained from https://github.com/nytimes/covid-19-data/blob/master/us.csv for 161 days, from 21 January until 28 June 2020, including total daily tests as well as daily positive tests and deaths (Fig. 1A and B). In addition, data from New York City was obtained from https://github.com/nychealth/coronavirus-data from 3 March to 27 June 2020, and LA data were obtained from http://dashboard.publichealth.lacounty.gov/covid19_surveillance_dashboard/ from 10 March until 29 June 2020. It is important to note that the NYC and LA data sets back-date cases and deaths to their episode date. However, other sources, such as the New York State or California dashboards, show the data by report date. The U.S. data reflect reporting date as well. We truncated each mortality time series by removing the first few days, in which data were sparse. A power spectrum analysis on the raw data were performed using MATLAB (https://www.mathworks.com/help/matlab/ref/rand.html) fast Fourier transform analysis (8), a standard method for analyzing periodicity by converting a time series to the frequency domain. To test temporally localized oscillatory behavior, we performed wavelet analysis, but this analysis resulted in no indication of such time-dependent periodicity. We detrended the data by performing a 7-day rolling average on each time series in order to smooth the data and then dividing the original time series by its smoothed version to obtain a normalized time series which measures deviation from the smoothed data, thus capturing the short-term variations in the data while controlling for the longer-term trend. We then performed regression and correlation analyses on the normalized time series, comparing the normalized daily positive tests to normalized daily total tests.
