# Supplementary figures and images for: Oscillations in U.S. COVID-19 Incidence and Mortality Data Reflect Diagnostic and Reporting Factors
Source: mSystems. 2020 Jul 14;5(4):e00544-20. doi: 10.1128/mSystems.00544-20 (PMC7363007; doi:10.1128/mSystems.00544-20)

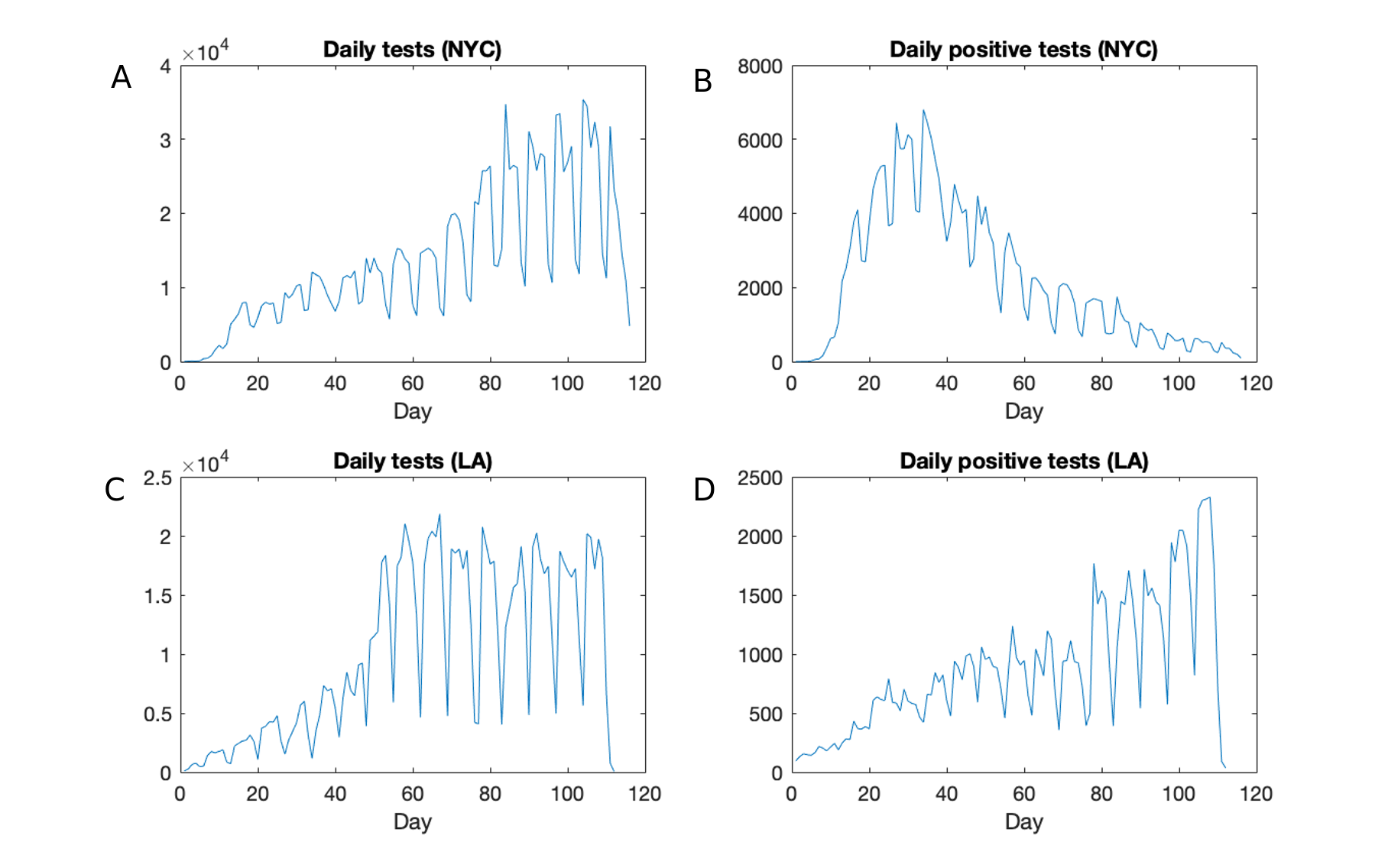

Supplement: FIG S1 [file mSystems.00544-20-sf001.tif]

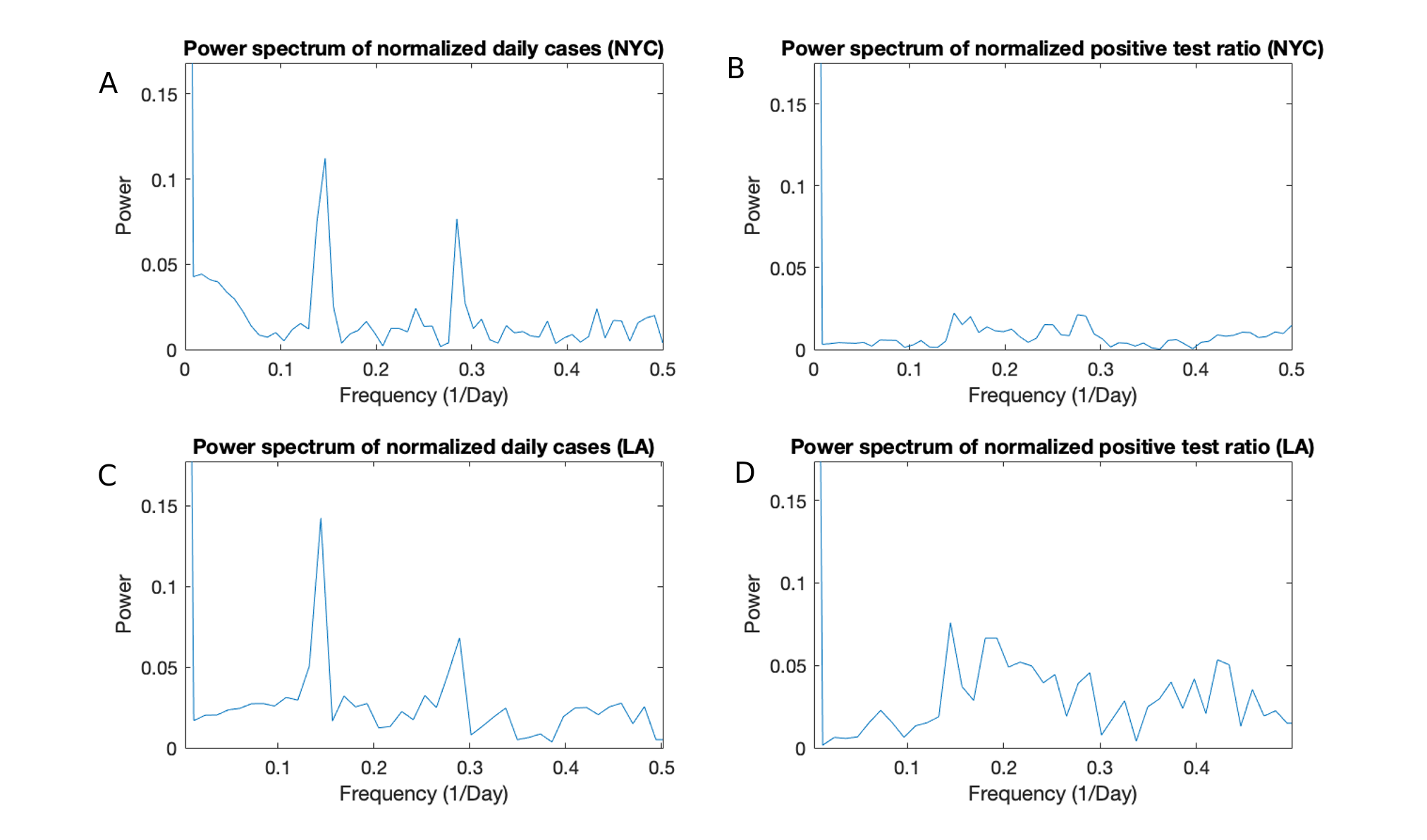

Supplement: FIG S2 [file mSystems.00544-20-sf002.tif]
